# Conduite à tenir devant un utérus didelphe associé à un hémivagin borgne

**Published:** 2012-11-18

**Authors:** Fatima Zohra Fdili Alaoui, Hakima Bouguern, Sofia Jayi, Nadia Squalli, Moulay Abdilah Melhouf

**Affiliations:** 1 Service de gynécologie-obstétrique II, CHU Hassan II, Fès, Maroc; 2 Service de radiologie, Chu Hassan II, Fès, Maroc

**Keywords:** Utérus didelphe, hémivagin borgne, hématocolpos, diagnostic, traitement chirurgical, pronostic, Uterus didelphys, blind hemivagina, Hematocolpos, diagnosis, surgical treatment, prognosis

## Abstract

L'utérus didelphe avec hémivagin borgne est une malformation rare, souvent diagnostiquée juste après les premières règles. La survenue d'un hématocolpos associée à une hématométrie et parfois un hématosalpinx est responsable de douleurs pelviennes et d'une dysménorrhée de plus en plus invalidante. Le diagnostic est posé par l’échographie pelvienne et selon l'urgence par l'imagerie par résonnance magnétique; l'agénésie rénale ipsilatérale est constante dans ce type de malformation. Le traitement consiste en une résection large de la cloison vaginale permettant ainsi un drainage continu de l'hémi utérus rétentionnel associé à une cœlioscopie objectivant les répercussions tubaires et pelviennes. Nous rapportons un cas d'utérus didelphe avec hémivagin borgne diagnostiqué à l’âge de 23 ans, nous discuterons à travers ce cas les aspects cliniques, diagnostiques et thérapeutiques de cette malformation utérine.

## Introduction

L'incidence réelle des malformations utérines est difficile à apprécier dans la littérature. Si les hypoplasies utérines et la présence des éperons en font partie, cette incidence varie autour de 6 - 7% dans la population des femmes fertiles et plus de 25% des femmes avec des antécédents de pertes fœtales répétées. Les utérus didelphes avec hémi-vagin borgne sont rares se manifestant le plus fréquemment lors des premières menstruations par un hématocolpos unilatéral avec hématométrie voire hématosalpinx responsable de dysménorrhée primaire et d'algies pelviennes [1]. Le diagnostic est posé par échographie pelvienne complétée par résonnance magnétique. Nous rapportons un cas d'utérus didelphe avec hémivagin borgne diagnostiqué à l’âge de 23 ans, nous discuterons à travers ce cas les aspects cliniques, diagnostiques et thérapeutiques de cette malformation utérine.

## Patient et observation

Madame ND, 23 ans, nulligeste mariée depuis 1 an, consulte pour douleurs pelviennes chroniques surtout gauches apparues depuis 1 an sans irradiation, associées à des dysménorrhées de plus en plus invalidantes sans troubles du cycle ni troubles urinaires ou digestifs.

**Figure 1:**
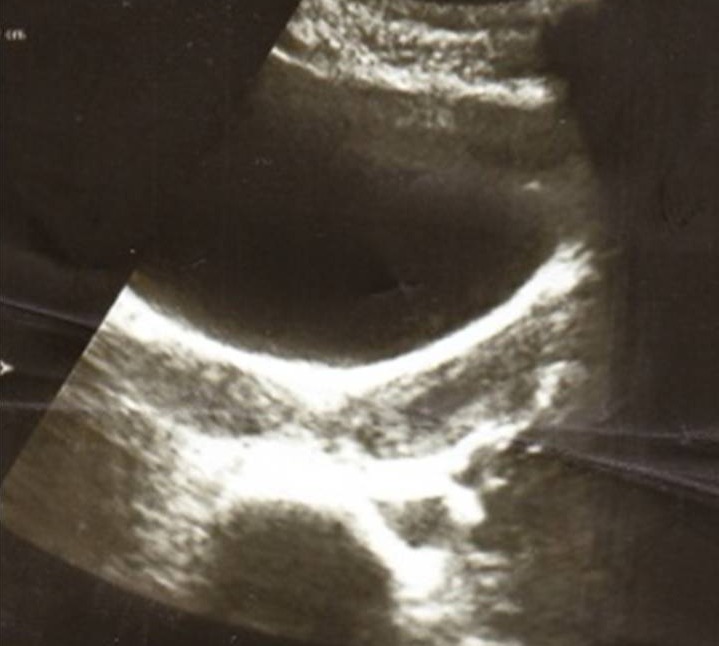
Aspect échographique de l'uterus de la patiente en coupe transversale montrant deux hémi matrices utérines avec petite hématométrie gauche

**Figure 2:**
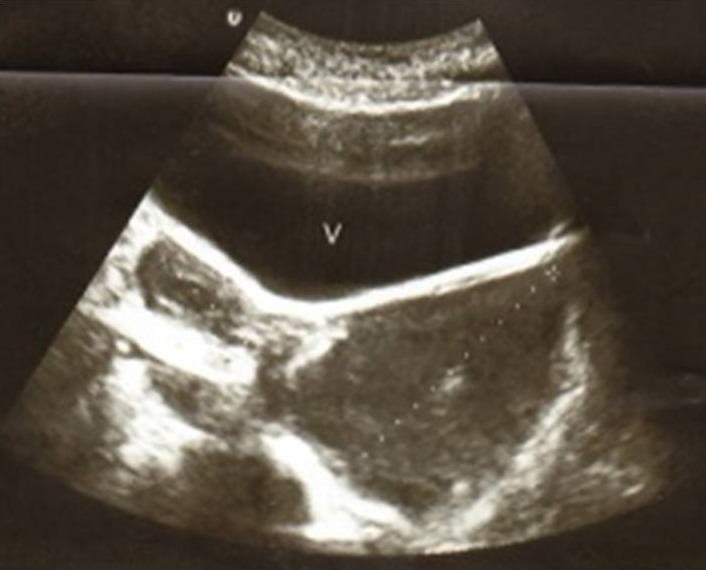
Aspect échographique montrant une hémimatrice gauche communiquant avec une image hypoéchogène vaginale (82mm) en faveur d'un hématocolpos gauche

A l'examen gynécologique, on note l'absence de visualisation de pertuis cervical avec au toucher vaginal et rectal un empâtement sensible vaginale gauche. A l’échographie abdominopelvienne (Figure 1, Figure 2): visualisation de deux hémimatrices utérines dont la gauche présente une petite hématométrie communiquant avec une volumineuse masse hypoéchogène faisant 82 mm suspectant un hématocolpos gauche. Une agénésie rénale gauche a été aussi objectivée.

**Figure 3:**
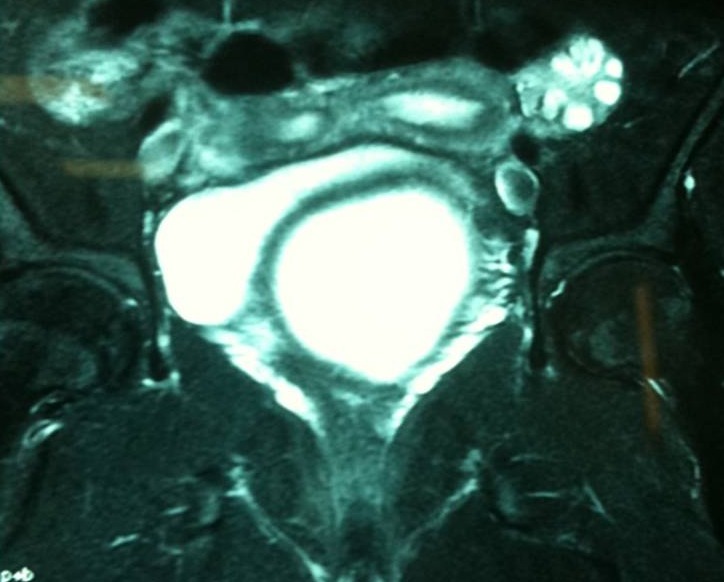
Un aspect d'imagerie par résonnance magnétique en faveur d'un utérus bicorne bicervical avec hématocolpos sur hémivagin borgne

**Figure 4:**
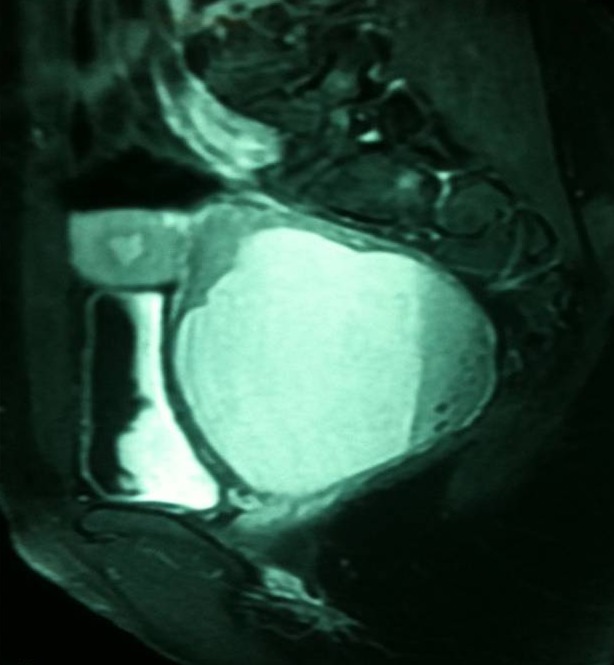
Un autre aspect d'imagerie par résonnance magnétique en faveur d'un utérus bicorne bicervical avec hématocolpos sur hémivagin borgne

**Figure 5:**
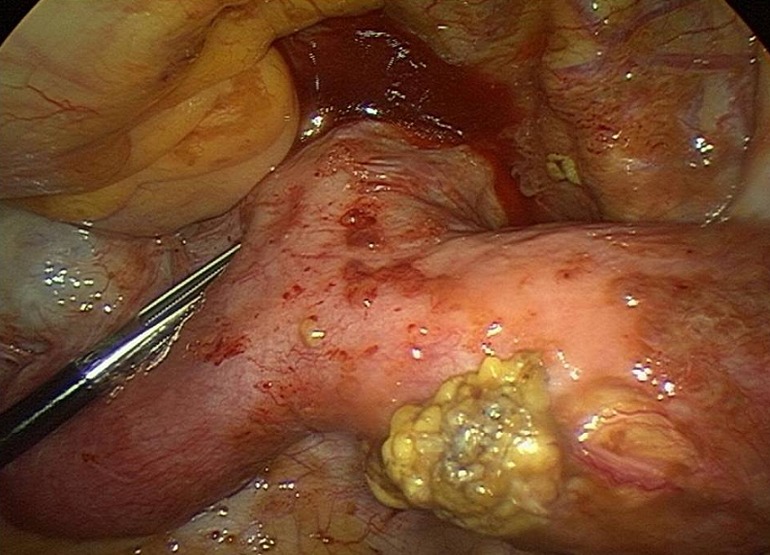
Aspect coelioscopique confirmant le diagnostic d'un utérus bicorne

L'imagerie par résonnance magnétique (Figure 3, Figure 4) montre un aspect en faveur d'un utérus bicorne bicervical, avec hémivagin borgne et hématométrie gauche. La coelioscopie confirme la malformation utérine (Figure 5). L'hémivagin borgne est incisé par voie basse et la résection d'une colerette vaginale est effectuée laissant apparaitre le deuxième col. Un capitonnage est réalisé par du fil résorbable vicryl numéro 1.

La patiente est mise sous amoxicilline protégée, à j4 on note une surinfection du site de capitonnage avec issue de leucorrhées verdâtres dont le prélèvement est en faveur de *Klebsiella pneumoniae*; L’évolution est favorable sous céphalosporines 3ème génération.

## Discussion

L'utérus bicorne est une malformation utérine liée à l'arrêt de l'organogénèse entre 10 et 12 semaines de grossesse, avec anomalie de fusion des deux canaux de Müller [1]. Selon la classification de Musset [2], il faut distinguer: **Utérus bicornes unicervicaux**: ils correspondent à deux hémiutérus fusionnés à une partie basse avec un col unique et selon les cas un isthme unique (pseudounicornes) ou deux hémi-isthmes indépendants. La séparation commence au dessus de la mi-hauteur théorique du corps utérin; **utérus bicornes bicervicaux**: ou les canaux de Muller gardent leur dualité sur toute la hauteur de l'organe. L'utérus didelphe est la variété où les deux cols et les deux vagins sont séparés nettement alors qu'ils sont accolés dans les autres variantes. Trois entités sont à distinguer: Utérus bicorne bicervical; Utérus bicorne bicervical avec hémivagin borgne et Utérus bicorne bicervical avec vagin borgne perméable cloisonné ou non.

Les utérus bicornes bicervicaux correspondent à la classe 3 selon la classification de l'American Fertility society, et de Buttram, et au type3 de la classification d'Acien. Les malformations de l'appareil urinaire apparaissent au même moment quand les canaux de Wolf et de Müller sont topographiquement proches; L'agénésie rénale, rapportée dans plusieurs séries est presque constante [3].

Dans le cas d'un utérus bicorne bicervical, le défaut de canalisation du bourgeon vaginal d'un coté est à l'origine d'un hémivagin borgne, lequel, lors de la ménarche explique le développement d'un hématocolpos unilatéral et par reflux du sang dans la cavité utérine voire parfois dans la trompe d'une hématométrie et d'un hématosalpinx responsable de dysménorrhée primaire et d'algies pelviennes. L'examen gynécologique dont le toucher rectal s'attache à retrouver le bombement de l'hémivagin rétentionnel dans la filière génital [4].

Les examens radiologiques apportent la confirmation diagnostique et recherchent les complications associées. L’échographie pelvienne par sa facilité d'accès reste le moyen le plus adapté et le moins invasif pour assoir le diagnostic rapidement en mettant en évidence la bifidité de l'appareil génital, elle apprécie le volume rétentionnel dans le vagin, l'utérus voir la trompe; Elle recherche aussi une agénésie rénale ipsilatérale associée. L’échographie tridimentionnelle est intéressante par la représentation spatiale de l'anomalie avec visualisation des rapports anatomiques [5]. En dehors de la situation d'urgence, l'imagerie par résonnance magnétique (IRM) est l'examen de choix pour réaliser le diagnostic différentiel: on parlera d'utérus septus complet quand le corps utérin est unique et qu'une cloison descendant jusqu’à l'endocol sépare deux cavités endométriales. On évoquera le diagnostic d'un utérus didelphe quand les deux corps utérins sont distincts l'un de l'autre avec deux filières endocervicales également. L'hémi vagin borgne pourra aussi être suspecté par l'IRM en plus de l'examen clinique [4, 6].

Quand le diagnostic est évoqué tardivement, on recherchera une fistule ayant permis un drainage progressif mais insuffisant de la rétention vers le coté perméable; l'examen objectivera un écoulement purulent en latéro cervical. Si la fistule se situe au niveau cervical, seule l'hystérographie permet de la mettre en évidence. L'hystéroscopie peropératoire peut difficilement visualiser le trajet fistuleux [7].

Le traitement chirurgical comporte une résection large de la cloison vaginale ceci dans le but d'assurer un drainage de l'hématocolpos et d’éviter la sténose vaginale secondaire. En effet, quand le drainage est simple sans résection de la collerette vaginale, l’évolution se fera vers la fibrose et la sténose vaginale. Le contrôle échographique en peropératoire s'assure de la bonne vidange de l'hémiutérus malformé [8].

La coelioscopie confirme le diagnostic mais aussi évalue les conséquences de la rétention utérovaginale: le reflux sanguin est responsable de réaction inflammatoire pelvienne à l'origine d'adhérences tubo-ovariennes et le développement d'un hydrosalpinx qui se surinfecte par la suite. L'endométriose péritonéale est une autre conséquence du reflux menstruel avancée par plusieurs travaux [9].

Le pronostic immédiat après le traitement chirurgical est satisfaisant avec disparition des douleurs pelviennes [10]. Les chances de procréation de ces patientes sont préservées; la rétention utérine même de longue durée n'altère ni l'endomètre ni la possibilité d'implantation. La survenue de fausses couches et de grossesses extra-utérines par altérations tubaires associées est majorée. Au cours de la grossesse estimée à haut risque, ces patientes sont plus exposées au risque d'accouchement prématuré par réduction de la taille de la cavité utérine, de présentation vicieuse et de dystocies au cours du travail occasionnant une augmentation du taux des césariennes [4].

## Conclusion

L'utérus bicorne bicervical avec hémivagin borgne est une malformation rare, responsable dès les premières ménarches d'algies pelviennes et de dysménorrhées de plus en plus invalidantes. L'association de l’échographie pelvienne et (en dehors de l'urgence) de l'imagerie par résonnance magnétique qui reste l'examen de choix confirme le diagnostic. Le traitement est chirurgical consistant en une résorption complète de la cloison vaginale permettant le drainage continu de la rétention menstruelle et évitant la fibrose et la sténose vaginale en post-opératoire. Les chances de procréation sont préservées, avec toutefois un risque majoré de fausses couches et de grossesses extra-utérines. Lors d'une grossesse, le risque de prématurité et de présentations dystociques reste accru.
